# A Profile of eHealth Behaviors in China: Results From a National Survey Show a Low of Usage and Significant Digital Divide

**DOI:** 10.3389/fpubh.2018.00274

**Published:** 2018-09-26

**Authors:** Y. Alicia Hong, Zi Zhou

**Affiliations:** ^1^School of Public Health, Texas A&M University, College Station, Texas, TX, United States; ^2^School of Public Health, Xiamen University, Xiamen, China

**Keywords:** eHealth behavior, national survey, digital divide, Health informatics, China

## Abstract

**Background:** The widely accessible Internet has boosted an enthusiasm for eHealth in China, but we know little about eHealth behaviors in the general population.

**Objective:** To assess the prevalence of eHealth behaviors in general Chinese population and identify the predictors of digital divide.

**Methods:** A nationally representative survey was administered in 2016–2017 with a sample size of 4,043. Five eHealth behaviors were assessed: search health information, communicate with healthcare providers, connect with patients of similar health conditions, buy medicine, and make doctor's appointment online. Multivariate logistic regression was employed to assess the independent relationship between eHealth behaviors and key demographic variables.

**Results:** About 33% of participants have ever searched health information online, and the prevalence of other eHealth behaviors was less than 10%. The adoption of eHealth behaviors was significantly associated with younger age, more education, higher income, and urban residence. By contrast, gender, employment status, health insurance, and health status were not associated with eHealth behavior.

**Conclusion:** The adoption of eHealth behaviors in the general Chinese population was low, and a significant digital divide exists. We caution against the speedy development of Internet hospitals and call for more resources allocated to bridge digital health divide.

## Introduction

The healthcare system in middle- and low-income countries continue to face considerable challenges in providing accessible, affordable, and quality health services to a large population. The rapid penetration of Internet and mobile tools offers a promising solution to address these mounting challenges. Despite the growing enthusiasm and evidence base, recent systematic reviews suggested that the quantity and quality of eHealth programs are still limited, and few pilot programs have been scaled-up or translated into sustainable policies (1).

As the most populous developing country, China is facing such mounting challenges as limited healthcare resources, unequal access to healthcare, substantial rural-urban health disparities, and an aging population. The massive healthcare reform since 2009 was aimed to address these challenges (2). China also hosts the largest population of netizens. As of 2016, China had 731 million Internet users (penetration rate 53%) and 1.3 billion mobile phone users (penetration rate of 90%) (3). The Chinese government is encouraging all healthcare agencies to utilize China's robust telecommunication infrastructure to assist in national healthcare reform initiatives; an increasing number of government-sponsored Internet hospitals and eHealth interventions have been established (4–6). However, a closer look at the data from the burgeoning eHealth initiatives revealed that most eHealth interventions were pilot projects conducted in major metropolitans; (5, 6) and the surveys on attitudes toward eHealth programs were typically collected from medical professionals or based on small convenience samples (7, 8). Data are missing on the readiness of eHealth adoption in the general Chinese population.

The literature from developed countries such as the U.S. suggested that access to mobile tools is not equivalent to using mobile tools for health purposes; and despite ubiquitous access to Internet, a persistent digital divide persists (9). Within such a complex context of high enthusiasm of Chinese government to promote Internet hospitals and limited global evidence of eHealth program sustainability from middle- and low-countries (1, 5), understanding eHealth behaviors of the Chinese population is an essential first step to formulate evidence-based eHealth policies. The current study aimed to fill up this literature gap by presenting a profile of eHealth behaviors in the general Chinese population with two research questions: (1) what's the prevalence of eHealth behaviors in China, and (2) what are the factors associated with adoption of eHealth behaviors?

## Methods

### Data source

The survey was conducted through the research project of China Governance and Public Policy Surveys (CGPPS), collaborated by the Texas A&M University (TAMU) in US and Southwestern University of Finance and Economics (SWUFE) in China from 2016 to 2017. The CGPPS employed a three-stage stratified probability proportion to size (PPS) random sample design with additional onsite GPS/GIS remote sensing sampling strategy to draw a representative sample of Chinese adults aged 18 and older in mainland China (excluding Tibet, Hong Kong, and Macau). A total 6,682 respondents were drawn and contacted, and 4,043 interviews were completed, yielding a final response rate of 60.50%. The survey was administered through random dialing Computer Aided Telephone Interviewing (CATI) system. All data were double entered and quality checked. The study protocol was approved by the Institutional Review Boards in TAMU and SWUFE.

### Measures

The 5 measures of *eHealth behaviors* were adopted from the US National Cancer Institute's Health Information National Trends Survey (HINTS). Since its inception in 2003, the biennial survey of HINTS has been considered as the leader in measuring eHealth behaviors (10). The measures included: (1) search health information online, (2) buy medicine online, (3) communicate with healthcare providers online, (4) connect with patients of similar health conditions online, and (5) make doctor's appointment online. *Demographic characteristics* included age, gender, education, rural/urban residence, employment status, income, and health insurance. In this study, education was transformed into an ordinal variable of 5 categories: no schooling or primary school, middle school, high school or vocational school, and college or above. Income was measured per capita income and was log-transformed for its skewness of distribution. *Health outcome* was measured by self-reported health; and the responses were dichotomized to good health (good or very good) and poor health (fair, poor, or very poor).

### Data analysis

We first analyzed the distribution of the five eHealth behaviors, then we used Chi-square (for categorical variables) and *t*-test (for continuous variables) to examine the relationship between each eHealth behavior and demographic variables. We then used multivariate logistic regression to examine the independent relationship between eHealth behavior and key demographics. We used STATA 12.0 for data analysis; SVY procedures were used to account for complex sampling design in estimating standard errors and incorporate sampling weights to inferential statistics.

## Results

The survey data revealed that 51% of the 4,043 participants had used Internet. However, only 33.3% of participants had searched health information, 9.4% bought medicine online, 8.2% communicated with peers of similar conditions, 10.0% communicated with doctors, and 10.1% made doctor's appointment online. Table 1 presents the independent association of key demographics and each eHealth behavior. Specifically, younger age, more education, higher income, and urban residence were significant predictors for all eHealth behaviors. The most significant predictor was rural/urban residence; urban residents were about twice more likely to engage in eHealth behaviors than their rural counterparts. On the other hand, gender, employment status, health insurance, and health status were not associated with any eHealth behavior.

**Table 1:** Independent association of eHealth behavior and key demographics (*n* = 4,043).

	**Search health info online**	**Buy medicine online**	**Communicate with patient peers online**	**Communicate with doctors online**	**Make appointment online**
Use^a^	33.3%	9.4%	8.2%	10.0%	10.1%
Independent variable	aOR^b^ (95% CI)^c^	aOR (95% CI)	aOR (95% CI)	aOR (95% CI)	aOR (95% CI)
Age	0.94^***^ (0.94–0.95)	0.96^***^ (0.95–0.97)	0.97^***^ (0.96–0.97)	0.95^***^ (0.94–0.96)	0.95^***^ (0.94–0.96)
Gender(female vs. male)	0.95 (0.81–1.13)	1.07 (0.84–1.35)	1.14 (0.89–1.45)	1.04 (0.83–1.30)	1.23 (0.98–1.56)
Education^d^	2.03^***^ (1.85–2.24)	1.48^***^ (1.29–1.70)	1.35^***^ (1.17–1.55)	1.37^***^ (1.20–1.56)	1.70^***^ (1.48–1.95)
Per capital income	1.25^***^ (1.16–1.35)	1.24^***^ (1.11–1.38)	1.14^*^ (1.02–1.28)	1.16^**^ (1.05–1.29)	1.36^***^ (1.21–1.52)
Residence (rural vs. urban)	0.54^***^ (0.45–0.65)	0.63^**^ (0.46–0.85)	0.54^***^ (0.39–0.74)	0.62^***^ (0.46–0.82)	0.52^***^ (0.38–0.71)
Employment	0.89 (0.74–1.09)	0.91 (0.70–1.20)	1.22 (0.92–1.63)	0.92 (0.71–1.19)	0.98 (0.74–1.28)
Have insurance	1.20 (0.88–1.64)	1.31 (0.84–2.06)	1.03 (0.66–1.60)	1.08 (0.72–1.63)	1.10 (0.72–1.70)
Health status (good vs. poor)	1.07 (0.91–1.26)	1.08 (0.85–1.36)	0.95 (0.75–1.21)	0.94 (0.75–1.18)	0.82 (0.67–1.03)

a *All percentage was calculated based on total sample of 4,043*.

b *aOR, Adjusted odds ratio*.

c *95% CI, 95% confidence interval*.

d *Education was created as an ordinary variable consisting of no schooling, primary school, middle school, high school, and college or above*.

*** *P < 0.001*.

## Discussion

Our findings suggested that access to Internet was not equivalent to eHealth behaviors, with 51% population have used Internet, only 33% Chinese have searched health info online and 10% used online healthcare communication or management. Chinese eHealth behaviors were strongly predicted by socio-economic status (SES), indicating a significant digital divide. The eHealth behavior adoption rates in China were lower than those in the US (10~33 vs. 18~57%) (11), but the patterns of persistent digital divide were similar (9); except that a significant rural-urban divide exists in China.

This study is limited in the following aspects. First, like other national surveys, potential volunteer and sampling biases might exist. However, our sampling scheme and data collection methods have been used in other credible national surveys; the response rates and participants demographic characteristics were similar to other national surveys (3, 10). Second, we only measured five eHealth behaviors and might have missed other important eHealth behaviors. More than 90% Chinese own a cell phone (3), how they used mobile apps or cell phones for health purposes needs further study. And finally, the cross-sectional nature of the study design prohibits interpretation of causal relationship.

Despite these limitations, the current study reports the first national survey on eHealth behaviors in China and one of the first from middle- and low-income countries. The low adoption rates of eHealth behaviors and significant digital divide suggested that the general Chinese population might not yet be ready for the “Internet hospitals” or “intelligent healthcare” (4, 5). Literature from high-income countries suggested that the ubiquitous Internet access has created a persistent digital divide and the eHealth movement has exacerbated health disparities (12). A recent survey on acceptance of Internet hospitals in some urban residents found that a majority of Chinese were reluctant to use such a system; instead, their primary concerns were long waiting time and difficulties of making a doctor's appointment (8). Our data of low prevalence of eHealth behaviors in the general population corroborated with such observations.

To conclude, we share the optimistic vision of eHealth systems in China, but low adoption of eHealth behaviors and the significant digital divide as evidenced in this study underscore the insufficient readiness of Internet hospitals in the general public. Hefty investment on Internet hospitals which may benefit only the young, healthy, educated, and wealthy urban residents, and further aggregate health disparities. We call for more resources for healthcare infrastructure building in the underserved communities and reduction of the digital health divide.

## Ethics statement

The study protocol, including the national survey protocol, was approved by the Institutional Review Boards in the Texas A&M University in US and the Southwestern University of Finance and Economics in China.

## Author contributions

YAH conceptualized and designed the study. ZZ analyzed the data. YAH drafted the manuscript and ZZ contributed in revision.

### Conflict of interest statement

The authors declare that the research was conducted in the absence of any commercial or financial relationships that could be construed as a potential conflict of interest.
